# Long-term Effect of Radiotherapy in Rectal Cancer Patients with Mucinous Tumor: A Large Population Based Study

**DOI:** 10.1038/srep43821

**Published:** 2017-03-08

**Authors:** Xu Guan, Senhao Jia, Wei Chen, Zheng Jiang, Zheng Liu, Yinggang Chen, Guiyu Wang, Xishan Wang

**Affiliations:** 1 Department of Colorectal Surgery, The Second Affiliated Hospital of Harbin Medical University, Harbin, China; 2 Department of Colorectal Surgery, Cancer Institute & Hospital, Chinese Academy of Medical Sciences, Peking Union Medical College, Beijing, China; 3 Department of Vascular and Endovascular Surgery, Chinese PLA General Hospital, Beijing, China; 4 Follow up center, The Second Affiliated Hospital of Harbin Medical University, Harbin, China

## Abstract

Due to distinct biological behavior of mucinous adenocarcinoma (MAC) and signet ring cell cancer (SRC), the efficacy of radiotherapy on long-term outcome for rectal cancer (RC) patients with mucinous tumors is still unclear. Here, we identified 1808 RC patients with MAC/SRC from Surveillance, Epidemiology, and End-Results (SEER) database from 2004 to 2013. Patients were divided into two subgroups according to different therapeutic strategies, including surgery alone and surgery combined with radiotherapy. Kaplan–Meier methods and Cox regression models were used to access the influence of therapeutic strategy on long-term survival outcomes. The 5-year and 10-year cancer specific survival (CSS) were improved in stage II and III patients who underwent surgery and radiotherapy compared with patients who underwent surgery alone. These results were further confirmed following propensity score matching. In addition, radiotherapy was deemed as independent good prognostic factor in patient with MAC/SRC. In subgroup analysis, the result also demonstrated that long-term survival was improved following radiotherapy. However, there was no prognostic difference between preoperative and postoperative radiotherapy. In conclusion, radiotherapy could improve survival for RC patients with MAC and SRC, but only for patients in stage II and III. This finding supported the application of radiotherapy in clinical practice.

Accumulating studies have shown that the administration of surgery combined with radiotherapy has significantly improved local control and decreased the incidence of local recurrences for patients with rectal cancer (RC)^1^,^2^,^3^,^4^. This combined treatment modality has been considered as standard therapy for patients with locally advanced RC. Despite the recommendation of radiotherapy to RC patients, the tumor response and prognostic outcome are variable to radiotherapy^5^. The histological type of RC may play a vital role for this variation^6^.

Most commonly studied subtypes of RC include mucinous adenocarcinoma (MAC) and signet ring cell cancer (SRC). Both histologies are differentiated from standard adenocarcinoma by levels of mucin within the tumor. MAC is defined as a tumor possessing more than 50% of the lesion being composed of extracellular mucin produced by tumor acinar cells. SRC is another variant subtype that contains a large amount of intracytoplasmic mucin, which pushes the nucleus to the cell periphery. The pool of mucin in a signet ring cell mimics the appearance of a finger hole and thereby gives the characteristic appearance of a signet ring cell, these tumors presented with even worse prognosis in the category of mucin secreting tumor^7^,^8^.

It is well known that MAC and SRC represent distinct clinicopathological features and molecular pathways, which may contribute them to have a more advanced stage of disease, a worse prognosis and different therapeutic response compared with adenocarcinoma^8^,^9^,^10^,^11^,^12^. The purpose of this study is to estimate the long-term survival benefit of adjuvant radiotherapy in RC patients with MAC and SRC.

## Results

### Patient characteristics

A total of 1808 eligible RC patients with MAC/SRC were collected from the Surveillance, Epidemiology, and End-Results (SEER) database during the 10-year study period, including 1607 patients with MAC and 201 patients with SRC. Of the cohort, 452 patients (25.0%) received surgery alone, and 1356 patients (75.0%) underwent surgery combined with radiotherapy. The proportion of white was 80.9%, accounting for the majority of patients collected. For patients aged <70 years, they were more likely to receive radiotherapy compared with patients aged ≥70 years. In surgery combined with radiotherapy group, 62.4% of patients were in stage III, the proportion of patients in stage III was decreased to 40.9% in surgery alone group. Furthermore, 75.7% of patients obtained sphincter preserving surgery in surgery alone group; the counterpart decreased to 66.8% for patients who underwent radiotherapy. The detailed information was listed in Table 1.

**Figure 1:**
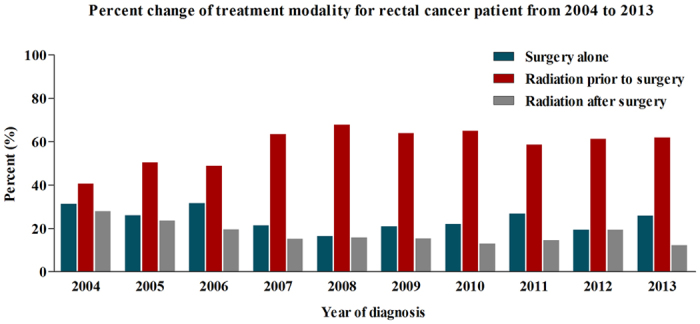
Trends of patient proportions according to different therapeutic strategies.

Here, we also observed the trend about different treatment strategies including surgery alone, radiation prior to surgery and radiation after surgery. The results showed that the increasing trend of radiation prior to surgery was observed as a whole from 2004 to 2013. The proportion of patients treated with radiation after surgery were gradually decreasing from 2004 to 2013 (Fig. 1). However, there was no obvious change of proportion in patients who underwent surgery alone.

### Survival comparisons between surgery alone group and surgery combined with radiotherapy group.

**Figure 2:**
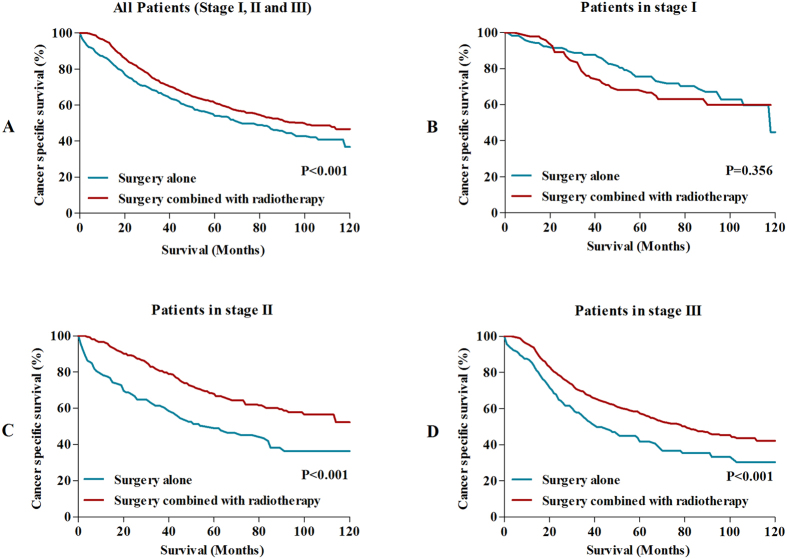
(**A**) Comparisons of CSS in all patients with MAC/SRC. (**B**) Comparisons of CSS in stage I patients with MAC/SRC. (**C**) Comparisons of CSS in stage II patients with MAC/SRC. (**D**) Comparisons of CSS in stage III patients with MAC/SRC.

With the aim of estimating whether RC patients with MAC/SRC could obtain survival benefit from radiotherapy, we compared 5-year and 10-year cancer specific survival (CSS) between patients receiving surgery alone and patients receiving surgery combined with radiotherapy. The results showed that 5-year CSS and 10-year CSS for patients treated with radiotherapy were 61.4% and 46.7%, which were significantly higher than patients received surgery alone (5-year CSS: 46.7%, 10-year CSS: 36.8%) (P < 0.001) (Fig. 2A). Furthermore, we separately analyzed the effects of radiotherapy on long-term survival in stage I, stage II and stage III. The results showed that patient in stage II and stage III could gain survival benefits from radiotherapy compared with patient who underwent surgery alone (Fig. 2C and D). However, there was no survival difference for patients in stage I between two groups (Fig. 2B).

**Figure 3:**
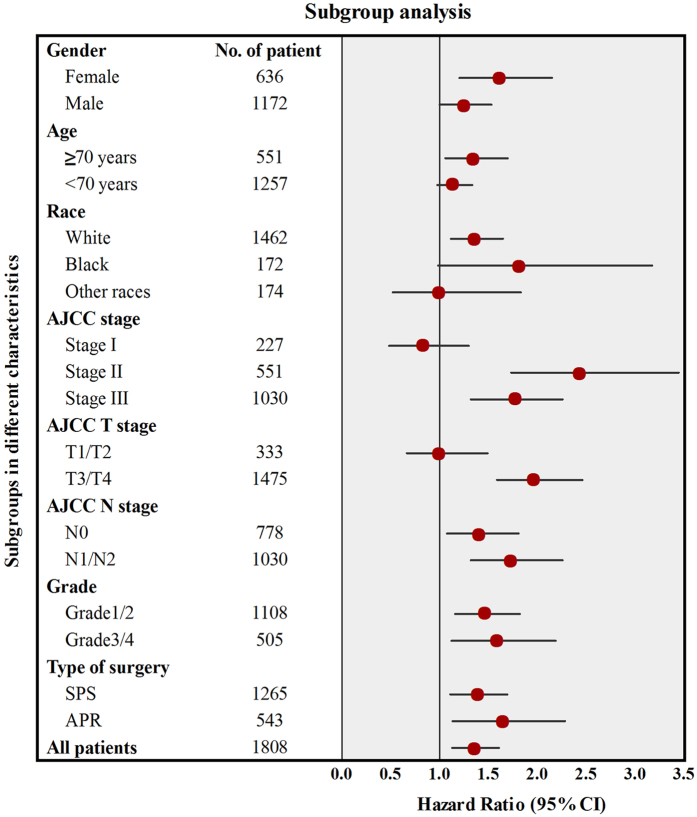
Survival comparisons between surgery alone group and surgery combined with radiotherapy group in subgroup analysis.

To further determine the prognostic consistency between two different treatment strategies, we divided patients into 18 subgroups based on each of different demographic and clinicopathological characteristics, and Cox’s regression model was separately used to estimate hazard rate (HR) and 95% confidence interval (CI) in each subgroup (Fig. 3). The results indicated that patients who underwent radiotherapy could obtain much more survival benefits than patients treated with surgery alone. The influence of treatment strategy with respect to CSS was homogeneous in 15 subgroups with HR more than 1.0, except for patients in stage I and patient in T1/T2. Therefore, this finding sufficiently established that patients treated with radiotherapy could obtain more survival benefit than patients receiving surgery alone.

### Survival comparisons following propensity score matching (PSM)

**Figure 4:**
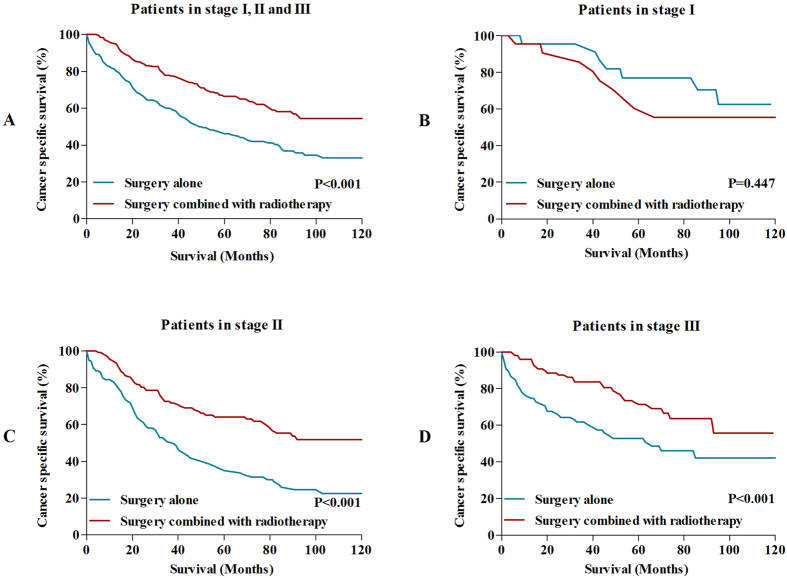
(**A**) Comparisons of CSS in patients with MAC/SRC after propensity score matching. (**B**) Comparisons of CSS in stage I patients with MAC/SRC after propensity score matching. (**C**) Comparisons of CSS in stage II patients with MAC/SRC after propensity score matching. (**D**) Comparisons of CSS in stage III patients with MAC/SRC after propensity score matching.

After PSM, there were totally 546 patients left, with 1:1 ratio in surgery alone group and surgery combined with radiotherapy group. The characteristics between two groups were well balance in the aspect of gender, race, TNM stage and surgical approach, with P > 0.05. All changes of characteristics after PSM were showed in Table 2. Then, the long-term survivals were compared between surgery alone group and surgery combined with radiotherapy group. The results showed that the 5-year CSS and 10-year CSS for patients who underwent surgery combined with radiotherapy were 66.4% and 54.5%, which were significantly higher than those who received surgery alone (5-year CSS: 46.2%; 10-year CSS: 33.1%) (P < 0.001) (Fig. 4A). In addition, we also compared the long-term survival in stage I, stage II and stage III, respectively (Fig. 4B–D). The results were similar to primary survival comparisons before PSM.

### Identifying adverse prognosis factors for RC patients with MAC/SRC

To further explore the factors that influenced long-term survival of patient with MAC/SRC, univariate and multivariate Cox regression analyses were performed to determine prognostic factors (Table 3). The results suggested that receiving surgery alone was considered as independent adverse prognostic factor for CSS in RC patients with MAC/SRC. In addition, other characteristics including stage II/III, stage N1/N2 and abdominoperineal resection were all identified as independent poor prognostic factors.

### Comparisons of long-term survival between patients receiving preoperative radiotherapy and patients receiving postoperative radiotherapy

**Figure 5:**
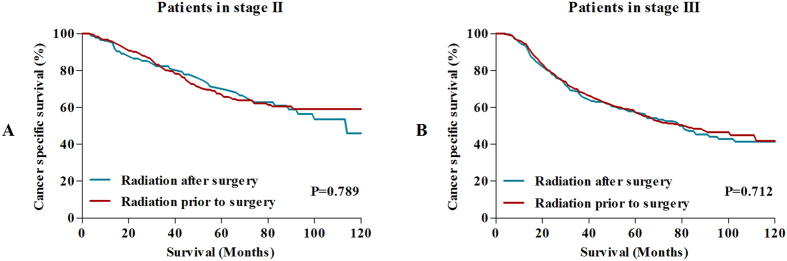
Survival comparisons between patients treated with preoperative radiotherapy and patients treated with postoperative radiotherapy.

With the aim of estimating the effect of therapy sequence on long-term survival, we compared the 5-year CSS and 10-year CSS between patients receiving preoperative radiotherapy and patients receiving postoperative radiotherapy. For patients in stage II and stage III, no survival differences were observed between two groups of patients (Fig. 5A and B).

## Discussion

Although MAC was first described in 1923, its clinicopathological and prognostic features remains a controversial entity currently. Some series have suggested that MAC and SRC were associated with worse prognosis of colorectal cancer^13^,^14^, but others have failed to find any difference of survival outcome compared with adenocarcinoma.

The American Joint Committee considered that MAC subtype was not one prognostic factor when matched with adenocarcinoma for similar stage and grade. The National Comprehensive Cancer Network (NCCN) guideline have not attributed to mucinous histology as a risk factor that affect the therapeutic decision. In addition, current clinical practice considered them similar to the non-mucinous tumors and histology does not influence therapeutic strategy making. However, lots of studies have suggested that the differences about treatment response and survival outcome were observed between MAC/SRC and adenocarcinoma. Previous studies have shown that long-term survival outcome was better in patients with non-mucinous tumors than patients with mucinous tumors, and the histology of MAC was considered as an independent prognostic factor for prognosis in RC patients^13^. Mucinous tumors presented with different oncogenic and molecular pathways which may make them respond differently compared to non-mucinous tumors^14^.

Recently, Simha *et al*. found that MAC of rectum presented with a poor response to preoperative chemo-radiotherapy in the consideration of higher incidence of positive resection margin, greater residual nodal disease and larger residual tumor. Furthermore, they found that MAC had more chance of peritoneal dissemination and distant metastasis during the preoperative treatment^7^. Sengul *et al*. have found MAC was associated with a poor response to preoperative chemo-radiotherapy for RC patients with MAC. Although the short-term effect of radiotherapy for MAC and SRC were important influential factors for treatment decision making, long-term survival outcomes after radiotherapy should be paid close attention. To our best knowledge, there was no related study evaluating the efficacy of radiotherapy on long-term outcome for RC patients with mucinous tumors based on the large population. In this study, we found that the long-term survival of patients treated with radiotherapy were obviously better than patients treated with surgery alone. Although preoperative radiotherapy could effectively decreased local recurrence rate and increased sphincter preservation rate compared with postoperative radiotherapy^15^,^16^,^17^, no differences of long-term survival were observed between patients who underwent preoperative radiotherapy and patients who underwent postoperative radiotherapy.

Although the strengths of this study including large sample size, PSM test and subgroup analysis, many limitations should be explained. First of all, local recurrence would also be one primary endpoint in this study, but the SEER lack recurrence data, which contribute to the local control benefit of radiotherapy couldn’t be analyzed^18^. Secondly, the SEER has no information regarding adjuvant chemotherapy for RC patients as well as detailed information associated with treatment compliance, toxicity and histopathologic features, such as angiolymphatic invasion and margin of resection. All these factors are presented with prognostic value in RC patients^19^. Thirdly, the regimen of radiotherapy for RC includes two fractionation schedules: short-course and long-course radiation. However, the SEER database is short of detailed information about this regimen. We therefore could not perform further analysis with respect to the effect of short-course and long-course radiation on RC patients with MAC and SRC.

In conclusion, although MAC and SRC are distinct subtypes of RC with different biological behavior, pathological feature and treatment response compared to adenocarcinoma, RC patients with MAC/SRC could obtain more survival benefit from surgery combined with radiotherapy than surgery alone. Therefore, this finding further strengthened that this combined treatment strategy could also be considered as standard approach for RC patient with MAC and SRC.

## Materials and Methods

### Data source

We identified the cancer cases from the SEER database. The SEER is an openly accessed database, which covers approximately 28% of the US population^20^. It includes the demographic, incidence and survival data from 17 population-based cancer registries. The authors could extract cancer cases and population data from SEER database. Data in the SEER database do not require informed patient consent, because they were anonymized and de-identified prior to release.

We have got permission to acquire the research data file in the SEER program by National Cancer Institute, USA and the reference number was 10249-Nov2015. All methods were performed in accordance with the relevant guidelines and regulations of SEER database. The study design was approved by the Ethics Committee of the Second Affiliated Hospital of Harbin Medical University.

### Study population

We obtained RC patients with MAC/SRC in stage I to stage III according to Site Recode classification. The collected patients were diagnosed from 2004 to 2013, because the seventh edition of AJCC TNM stage system was available in SEER database since 2004. All patients were pathologically diagnosed with the histology of MAC or SRC. The therapeutic strategies for RC patients included surgery alone and surgery combined with radiotherapy. Radiotherapy was further composed of radiotherapy prior to surgery and radiotherapy after surgery. Other clinical characteristics extracted from SEER database included age, gender, race, year of diagnosis, AJCC TNM stage, grade and surgical approaches. The surgical approaches included sphincter preserving surgery and abdominoperineal resection. The exclusion criteria were as follows: alive with no survival time and dead due to other causes.

### Statistical analysis

Firstly, we evaluated the differences in patient characteristics between surgery alone group and surgery combined with radiotherapy group using the χ^2^ test. The CSS was estimated, which was defined as the time from the RC diagnosis until cancer recurrence or metastasis, cancer-associated death and the end of follow up. The CSS was estimated with Kaplan-Meier method, and log-rank tests were used to compare the differences of CSS curves. Univariate and multivariate Cox’s regression model were performed to estimate HR and exact 95% CIs. Furthermore, patients were classified into different subgroups based on different characteristics. These subgroup analyses of CSS were separately performed using Cox regression model to determine the prognostic consistency between surgery alone group and surgery combined with radiotherapy group. All statistical tests were two sided, P < 0.05 was considered to be statistical significance. All statistical analyses were estimated using the statistical software package SPSS 20.0 (IBM Corp, Armonk, NY, USA) and R version 2.12.0 (www.r-project.org).

### PSM

A propensity 1:1 matched analysis was done to reduce possible bias to a minimum in this retrospective analysis. Propensity scores were calculated using logistic regression model for each patient in both surgery alone group and surgery combined with radiotherapy group. The covariates included in the regression were age, gender, race, AJCC TNM stage, AJCC T stage, AJCC N stage, grade and surgical approach. Patients in two groups were matched based on the propensity score. Covariates balance between two groups was examined by χ^2^ test. The survival comparisons were then performed for the propensity score-matched patients using the same methods as those in the primary analysis.

## Additional Information

**How to cite this article**: Guan, X. *et al*. Long-term Effect of Radiotherapy in Rectal Cancer Patients with Mucinous Tumor: A Large Population Based Study. *Sci. Rep.* **7**, 43821; doi: 10.1038/srep43821 (2017).

**Publisher's note:** Springer Nature remains neutral with regard to jurisdictional claims in published maps and institutional affiliations.

## Figures and Tables

**Table 1:** Characteristics among RC patients with MAC/SRC.

Characteristics	Surgery alone N = 452		Surgery combined with radiotherapy N = 1356		P
Age (Years)					<0.001
<70	225	49.8%	1032	76.1%	
≥70	227	50.2%	324	23.9%	
Gender					0.004
Male	268	59.3%	904	66.7%	
Female	184	40.7%	452	33.3%	
Race					0.480
Black	40	8.8%	132	9.7%	
White	374	82.8%	1088	80.3%	
Other	38	8.4%	136	10.0%	
Year of diagnosis					0.145
2004–2008	275	60.8%	772	56.9%	
2009–2013	177	39.2%	584	43.1%	
AJCC Stage					<0.001
Stage I	126	27.9%	101	7.4%	
Stage II	141	31.2%	410	30.2%	
Stage III	185	40.9%	845	62.4%	
AJCC T stage					<0.001
T1/T2	160	35.4%	173	12.8%	
T3/T4	292	64.6%	1183	87.2%	
AJCC N stage					<0.001
N0	267	59.1%	511	37.7%	
N1/2	185	40.9%	845	62.3%	
Grade					<0.001
Grade I/II	318	70.4%	790	58.3%	
Grade III/IV	105	23.2%	399	29.4%	
Unknown	29	6.4%	167	12.3%	
Surgical approach					<0.001
Sphincter preserving surgery	342	75.7%	906	66.8%	
Abdominoperineal resection	110	24.3%	450	33.2%	

**Table 2:** Characteristics among RC patients with MAC/SRC after propensity score matching.

Characteristics	Surgery alone N = 273		Surgery combined with radiotherapy N = 273		P
Age (Years)					0.028
<70	156	57.1%	181	66.3%	
≥70	117	42.9%	92	33.7%	
Gender					0.096
Male	157	57.5%	176	64.5%	
Female	116	42.5%	97	35.5%	
Race					0.809
Black	30	11.0%	27	9.9%	
White	217	79.5%	223	81.7%	
Other	26	9.5%	23	8.4%	
AJCC Stage					1
Stage I	24	8.8%	24	8.8%	
Stage II	142	52.0%	142	52.0%	
Stage III	107	39.2%	107	39.2%	
AJCC T stage					1
T1/T2	49	17.9%	49	17.9%	
T3/T4	224	82.1%	224	82.1%	
AJCC N stage					1
N0	165	60.4%	165	60.4%	
N1/2	108	39.6%	108	39.6%	
Grade					0.001
Grade I/II	206	75.5%	171	62.6%	
Grade III/IV	52	19.0%	67	24.5%	
Unknown	15	5.5%	35	12.8%	
Surgical approach					0.626
Sphincter preservation surgery	204	74.7%	199	72.9%	
Abdominoperineal resection	69	25.3%	74	27.1%	

**Table 3:** Univariate and multivariate analyses for RC patients with MAC/SRC.

Characteristic		Univariate analysis		Multivariate analysis	
		HR [95% CI]	P	HR [95% CI]	P
Gender	Female	1	0.728		
	Male	1.028 [0.879–1.203]			
**Age (Years)**	<70	1	0.179		
	≥70	1.115 [0.951–1.306]			
Race	White	1	0.380		
	Black	0.981 [0.755–1.276]			
	Others	1.190 [0.927–1.528]			
AJCC Stage	Stage I	1	0.015	1	0.037
	Stage II	1.455 [1.127–1.877]		1.743 [1.135–2.676]	
	Stage III	1.301 [1.018–1.662]		1.670 [1.113–2.507]	
AJCC T stage	T1/T2	1	0.006	1	0.292
	T3/T4	1.322 [1.084–1.613]		1.195 [0.858–1.665]	
AJCC N stage	N0	1	<0.001	1	<0.001
	N1/2	1.459 [1.250–1.702]		1.533 [1.300–1.809]	
Grade	Grade I/II	1	0.319		
	Grade III/IV	1.110 [0.937–1.314]			
Surgical approach	Sphincter preserving surgery	1	<0.001	1	<0.001
	Abdominoperineal resection	1.451 [1.242–1.696]		1.380 [1.177–1.618]	
Treatment strategy	Surgery alone	1	0.001	1	<0.001
	Surgery combined with radiotherapy	0.759 [0.644–0.895]		0.496 [0.404–0.608]	
